# Characterizing Cancer and Work Disparities Using Electronic Health Records

**DOI:** 10.3390/ijerph192315887

**Published:** 2022-11-29

**Authors:** Jaimi L. Allen, Ruofei Du, Thomas Powell, Khariana L. Hobbs, Benjamin C. Amick

**Affiliations:** 1 Department of Epidemiology, University of Arkansas for Medical Sciences, Little Rock, AR 72205, USA; 2 Department of Biostatistics, University of Arkansas for Medical Sciences, Little Rock, AR 72205, USA; 3 Department of Biomedical Informatics, University of Arkansas for Medical Sciences, Little Rock, AR 72205, USA; 4 Arkansas Department of Health, Fay W. Boozman College of Public Health, University of Arkansas for Medical Sciences, Little Rock, AR 72205, USA; 5 Winthrop P. Rockefeller Cancer Institute, University of Arkansas for Medical Sciences, Little Rock, AR 72205, USA

**Keywords:** employment, cancer survivorship, disparities

## Abstract

Advancements in cancer diagnosis and treatment have resulted in improvements in survivor outcomes; however, cancer survivors are more likely to experience adverse employment outcomes such as job loss, reduced working hours, and early retirement. The purpose of this study was to examine employment disparities among cancer survivors. Our study collected data from 29,136 cancer survivors (ages 18–65) between 2015 and 2021 using electronic health records (EHR) and linked to cancer registry data. Of those with employment information (n = 7296), differences in employment status were explored by race, ethnicity, sex, geography, marital status, education, age, and cancer site. Of the patients with employment status available, 61% were employed, 28% were not employed, 9% were disabled, 2% were retired. Logistic regression results revealed adjusted effects: a positive association between employment and marriage, while racial and ethnic minority adults, rurality, and certain age categories were less likely to be employed. Unadjusted results showed a positive association between employment and education. These results contribute to an emerging body of literature showing adverse employment outcomes for cancer survivors.

## 1. Introduction

The number of cancer survivors is growing rapidly due to advancements in screening and treatment [1]. Cancer survivors are defined as anyone who has ever been diagnosed with cancer regardless of where they are in their journey. [2] Today, the majority of cancer survivors are of working age [3] and choose to continue working throughout treatment [4]. Consequently, employment, as an outcome metric, is a critical indicator of quality of life and is important for cancer survivors, their family, and society as a whole [5,6]. Paid work is directly related to psychological and physical health outcomes, treatment recovery, and financial stability providing a feeling of normalcy and purpose [5,6]. While employment can provide substantial benefits to cancer survivors, they are more likely to become unemployed, retire early, and are less likely to be re-employed [7].

Health disparity populations, such as racial/ethnic minorities, underserved rural residents, and other populations with socioeconomic disadvantage [8] experience worse health and employment outcomes throughout their cancer journey. Racial and ethnic minority populations and other disparity groups struggle more with the behavioral, emotional, physical, and financial pressures associated with cancer and have a lower quality of life after receiving a cancer diagnosis [9,10]. Racial and ethnic minority cancer survivors are also less likely to return to work following a cancer diagnosis [11,12,13], contributing to income disparities. Compared to non-Hispanic white adults, racial and ethnic minority groups have higher rates of unpaid leave, shorter work weeks, loss of income, financial difficulties, job loss, loss of health insurance, and are more likely to stop treatment as a result [14,15,16,17].

Cancer survivors living in rural areas are more likely to experience fair or poor health and self-reported psychological distress and are more likely to retire early and endure health-related unemployment [18]. Rural communities have a higher poverty rate, lower education, less access to essential health services, and are more likely to be unemployed compared to those in urban areas [18,19]. Cancer survivors with lower education and income are also associated with higher levels of unemployment [20], and low-income and minority cancer survivors who remained working were less likely to work in accommodating work environments [12].

The purpose of the present study was to examine electronic health records (EHR) at a large medical center in a rural Southern state to investigate employment variations by race, ethnicity, geography, education, marital status, sex, age, and cancer site. The results of this study will provide critical foundational information on employment for cancer survivors, especially health disparity groups.

## 2. Materials and Methods

Data was obtained from over 29,000 electronic health records (EHR) housed in the Clinical Data Repository of a major academic health center. The Clinical Data Repository is a research data warehouse that includes EHR data from all UAMS sites throughout the state. Inclusion criteria were working-age adults (18–65 years old) who had been diagnosed with cancer from 2015 to 2021 and were not currently undergoing palliative care. The study was reviewed and approved by the University of Arkansas for Medical Sciences Institutional Review Board (Protocol 261945).

Initially, 63,966 records were obtained from the Clinical Data Repository on all patients who had a cancer diagnosis in their medical record. A total of 29,136 patients were included, and the most recent record for each patient was retained. The primary study outcome was employment status (n = 7296). Patients had the option of listing their employer on their medical records. These answers were then categorized into employed, retired, disabled, and not employed. Retired and disabled were further merged with not employed resulting in a dichotomous variable (yes/no) for employment status. The following variables were also collected from patient records: race, ethnicity, rurality, sex, education, marital status, age at diagnosis, and cancer type. Optional questions (employment and education) were the only variables with a high degree of missingness. Education (n = 599) was collapsed into five categories: some high school, high school graduate or received their general education development (GED) certification, completed some college, undergraduate degree, and graduate school or above. Racial categories were collapsed into three categories: White, Black, and Other. Rurality was determined based on the reported residential zip code at the time of the patient’s visit and was assessed using publicly available zip code approximations of census tract RUCA codes from the United States Department of Agriculture Economic Research Service (USDA-ERS) [21]. Cancer type was categorized based on ICD-10-CM codes for neoplasms [22].

Unadjusted and adjusted odds ratios (OR) and 95% confidence intervals (CI) from logistic regression analyses were calculated to estimate the association between employment and study variables (race, ethnicity, rurality, sex, education, marital status, age at diagnosis, cancer type). Multivariable linear regression was used to examine the influence of sociodemographic characteristics on employment. In preliminary analyses, all variables except for sex showed statistical (*p* ≤ 0.20) or conceptual importance and were retained in the final multivariable regression model. However, a decision was made to remove education from adjusted modeling due to considerable missingness. Cancer site was also removed from fully adjusted models due to sample size. In final adjusted models, a statistical significance level (*p* < 0.05) was used, and all statistically significant results were evaluated to determine if differences were clinically or practically significant. STATA version SE 16.1 (StataCorp LLC, College Station, TX, USA) was used for data analyses.

## 3. Results

A total of 7296 patients had available information on employment. The average age was 50.9 (±10.5) years. Most of the patients were employed (61%), female (77%), and white (66%). Approximately 40% of the patients held an associate degree or higher, 53% reported being married or having a significant other, and 25% lived in a rural area. Sociodemographics by employment status are shown in Table 1. 

Due to low representation of many cancer sites, cancer site was removed from adjusted modeling. Descriptive statistics in Table 1 show the following hierarchal tier of cancer sites beginning with the highest employment percentage: melanoma > neuroendocrine tumors > benign neoplasms > neoplasms of uncertain or unspecified behavior > thyroid and other endocrine glands > breast > male genital organs = mesothelial and soft tissue > in situ neoplasms > lip, oral cavity, and pharynx > urinary tract = bone and articular cartilage > lymphoid, hematopoietic, related tissue > female genital organs = eye, brain, other parts of the central nervous system > ill-defined, secondary, and unspecified neoplasms > respiratory and intrathoracic organs. 

As shown in Table 2, race, ethnicity, geography, marital status, age at diagnosis, and education were all statistically significant in unadjusted models. The following variables were positively associated with employment (more likely to be employed) in unadjusted models: married or having a significant other (reference category [ref]:single/widowed; OR 2.11 [95% CI, 1.90–2.36]), having an undergraduate degree (OR 2.33 [95% CI, 1.17–4.62]), and having a graduate degree or higher (ref: high school education; OR 7.32 [95% CI, 3.34–16.01]). The following variables were negatively associated with employment (less likely to be employed) in unadjusted models: Black (ref: White; OR 0.71 [95% CI, 0.64–0.79]), Hispanic (ref: non-Hispanic; OR 0.64 [95% CI, 0.48–0.85]), rural (ref: urban; OR 0.61 [95% CI, 0.55–0.68]), current age, and age at diagnosis. With a base category of 35–44 for age at diagnosis, ages 18–24 (OR 0.36 [95% CI, 0.27–0.49]), 45–54 (OR 0.79 [95% CI, 0.70–0.90]), and 55–65 (OR 0.64 [95% CI, 0.56–0.73]) were less likely to be employed. 

Education was not included in adjusted analyses due to missing data (n = 599); cancer site was not included due to small subgroup representation (i.e., 3 participants with malignant or secondary neuroendocrine tumors, 13 with bone and articular cartilage cancer). Adjusted analyses showed employment was only positively associated with being married or having a significant other (ref: single/widowed; OR 2.07 [95% CI, 1.84–2.33]). The following variables were negatively associated with employment: Black (ref: White; OR 0.78 [95% CI, 0.69–0.88]), Hispanic (ref: non-Hispanic; OR 0.53 [95% CI, 0.38–0.74]), rural (ref: urban; OR 0.56 [95% CI, 0.50–0.62]), and age at diagnosis. With a base category of 35–44 for age at diagnosis, ages 18–24 (OR 0.43 [95% CI, 0.32–0.58]), 45–54 (OR 0.77 [95% CI, 0.67–0.88]), and 55–65 (OR 0.61 [95% CI, 0.53–0.70]) were less likely to be employed.

## 4. Discussion

Overall, nearly 40% of cancer survivors in our study were not employed, which is relatively consistent with findings in larger studies. A meta-analysis conducted in 2009 included 36 studies and 20,366 cancer survivors found unemployment was 33.8% compared to 15.2% in healthy control participants [23]. In a retrospective cohort study in Taiwan with a follow-up period from 2004 to 2010, 48.9% of cancer survivors were not employed through 5 years post cancer diagnosis [24].

Analysis of results indicated being married or having a significant other was positively associated with employment. Other studies have found positive results with married cancer survivors calling it a “survival advantage” [25]. These marital benefits include better screening adherence [26], greater likelihood of being diagnosed at an earlier stage [27], better treatment [28], better survival [29], and lower mortality rates resulting from cancer [28]. Although married cancer survivors may experience a survival advantage [25], studies are mixed on the impact of marriage on employment. Having a partner can provide financial security potentially enabling the cancer survivor to stay at home or decrease working hours after diagnosis [30,31,32]. Adversely, having the partner’s emotional support can help facilitate a return to employment [33,34]. 

Study results also indicate higher education can be a predictor of employment. Although educational attainment was expected to be a significant finding in disparate populations, inconsistent documentation quality required this variable to be removed from the fully adjusted model. Higher educational attainment has been a predictor of employment for cancer survivors in previous literature [7,20,35]. However, other studies have found setbacks to educational achievement for younger cancer survivors, posing a potential survivor risk for these populations [36,37].

Study results support previous literature indicating racial and ethnic minority cancer survivors experience employment hardship compared to white counterparts [14]. A review of literature also shows that Black and Hispanic cancer survivors are also more likely to experience disproportionate levels of health insurance changes and income loss resulting in financial hardships and financial toxicity as a result of cancer and associated treatments [11,38]. Racial and ethnic minorities also have high levels of unpaid leave, reduced work hours, and are more likely to stop treatment, borrow money from friends or family, and skip medical and non-medical bills when compared to non-Hispanic white cancer survivors [14]. These experiences can decrease health-related quality of life and cause a delay or omission in necessary medical care [15]. Future studies should investigate the drivers behind these cancer disparities for racial and ethnic minorities to include discrimination and marginalization. 

Results indicated those under 25 years of age and over 44 years were less likely to be employed when compared to 35–44-year-olds. Similar results have been found by researchers investigating age at diagnosis. Two systematic reviews on employment in cancer survivors revealed cancer survivors over 44 years of age were more likely to be on continued sick leave and less likely to return to work after treatment [6,7]. Younger cancer survivorship has also been associated with adverse employment outcomes [39], indicating further research is needed to identify age-related employment disparities for cancer survivors so sufficient support is provided. 

Although descriptive statistics were included to show the employment percentage for each cancer site, cancer site was removed from adjusted modeling due to small sample size for some sites: neuroendocrine tumors, malignant and secondary (n = 3); bone and articular cartilage (n = 13); respiratory and intrathoracic organs (n = 37); mesothelial and soft tissue (n = 71); eye, brain, other parts of the central nervous system (CNS; n = 73); urinary tract (n = 85). Current literature has mixed results on cancer sites and employment. For instance, Park et al. found lung, brain/CNS cancer sites, and leukemia were associated with initial job loss, but those with leukemia, stomach, liver, and thyroid cancer were associated with re-employment [40]. A systematic literature review found the following cancer sites were associated with unemployment: liver, lung cancer, advanced blood and lymph malignancies, brain and CNS cancer sites, gastrointestinal cancers, pancreatic cancer, and head and neck cancers [7]. Chen et al. found patients with cervical cancer, female breast cancer, and thyroid cancer were most likely to be employed in the first and fifth year following diagnosis [24]. Further investigation into the association between employment and cancer type is recommended. 

### Strengths and Limitations

A study strength was the sample size. A large sample of patients included employment information even though this was not a required question to answer on health records (n = 7296). Due to considerable missing data concerning education (n = 599), it was subsequently removed from final adjusted models even though education had animpact on employment outcomes in unadjusted analyses. Cancer site was also excluded from final adjusted modeling due to low representation. Cancer staging and some treatment details were unavailable in de-identified data. Additionally, employment status was the only employment outcome available, so important information is unknown about work hours, work accommodations, and work functionality. Nearly all patients included in analyses had health insurance apart from 36 (0.5%) patients, so comparative analysis was not conducted. 

## 5. Conclusions

This study found marriage or having a significant other and higher levels of education to be significant predictors of employment for cancer survivors while risk factors associated with lower employment levels were race, ethnicity, rurality, age, and cancer site. These results can aid public health efforts to reduce the employment gap for cancer survivors. Future interventions should focus on populations most at risk for adverse employment outcomes so tailored approaches can be developed. Underestimating the scope and magnitude of the problem could result in additional health equity gaps.

## Figures and Tables

**Table 1:** Characteristics of cancer patients by employment status.

	Employed (n = 4430)	Not Employed (n = 2866)	Employment %	*p*-Value *
**Current age, years**				<0.001
18–24	11 (<1%)	21 (1%)	34%	
25–34	365 (8%)	267 (9%)	58%	
35–44	937 (21%)	457 (16%)	67%	
45–54	1242 (28%)	704 (25%)	64%	
55–65	1875 (42%)	1417 (49%)	57%	
**Age at diagnosis, years**				<0.001
18–24	88 (2%)	123 (4%)	42%	
25–34	663 (15%)	365 (13%)	64%	
35–44	1135 (26%)	575 (20%)	66%	
45–54	1423 (32%)	909 (32%)	61%	
55–65	1121 (25%)	894 (31%)	56%	
**Geography**				<0.001
Urban	3467 (78%)	1969 (69%)	64%	
Rural	963 (22%)	897 (31%)	52%	
**Sex**				0.73
Female	3320 (75%)	2158 (75%)	61%	
Male	1110 (25%)	708 (25%)	61%	
**Race**				<0.001
White	3068 (69%)	1786 (62%)	63%	
Black	1124 (25%)	924 (32%)	55%	
Other	238 (5%)	156 (5%)	60%	
**Ethnicity**				<0.01
Hispanic	95 (2%)	95 (3%)	50%	
Non-Hispanic	4335 (98%)	2771 (97%)	61%	
Education **				<0.001
Some high school (HS)	37 (9%)	47 (24%)	44%	
HS graduate/GED	19 (5%)	20 (10%)	49%	
Some college	44 (11%)	32 (16%)	58%	
College graduate	166 (41%)	75 (39%)	69%	
Grad school or above	139 (34%)	20 (10%)	87%	
**Marital status**				<0.001
Married/significant other	2670 (61%)	1169 (41%)	69%	
Divorced/separated	578 (13%)	597 (21%)	49%	
Single/widowed	1163 (26%)	1077 (38%)	52%	
**Cancer site (ICD-10-CM code)**				<0.001
Lip, oral cavity, and pharynx (C00–C14)	30 (<1%)	24 (<1%)	55%	
Respiratory and intrathoracic organs (C30–C39)	9 (<1%)	28 (<1%)	24%	
Bone and articular cartilage (C40–C41)	7 (<1%)	6 (<1%)	54%	
Melanoma and other MN of skin (C43–C44)	94 (2%)	45 (1%)	68%	
Mesothelial and soft tissue (C45–C49)	41 (<1%)	30 (1%)	58%	
Breast (C50)	320 (7%)	208 (7%)	61%	
Female genital organs (C51–C58)	134 (3%)	142 (5%)	48%	
Male genital organs (C60–C63)	55 (1%)	39 (1%)	58%	
Urinary tract (C64–C68)	46 (1%)	39 (1%)	54%	
Eye, brain, other parts of CNS (C69–C72)	35 (<1%)	38 (1%)	48%	
Thyroid and other endocrine glands (C73-C75)	98 (2%)	61 (2%)	62%	
Ill-defined, secondary, unspecified (C76–C80)	158 (3.6%)	199 (7%)	44%	
Neuroendocrine tumors—malignant and secondary (C7A–B)	2 (<1%)	1 (<1%)	67%	
Lymphoid, hematopoietic, related tissue (C81)	248 (6%)	245 (8%)	50%	
In situ neoplasms (D00–D09)	224 (5%)	166 (6%)	57%	
Benign neoplasms and benign neuroendocrine tumors (D10–D36, D3A)	2142 (48%)	1151 (40%)	65%	
Neoplasms of uncertain or unspecified behavior, polycythemia vera, and myelodysplastic syndromes (D37–D49)	787 (17.8%)	444 (15%)	64%	

Note: Percentages have been rounded and may not total 100%. * *p*-value of chi-squared test comparing difference of proportion between employed and not employed. ** Education is the only variable with missing values (n = 599).

**Table 2:** Odds ratios and 95% confidence intervals from logistic regression analyses for variables associated with employment in cancer survivors.

Variables	Unadjusted (n = 7296)			Adjusted (n = 7254)		
	OR	95% CI	*p*	OR	95% CI	*p*
Race (reference group: White)						
Black	0.71	0.64–0.79	**<0.01**	0.78	0.69–0.88	**<0.01**
Other	0.89	0.72–1.10	0.27	0.89	0.69–1.14	0.35
Ethnicity (reference group: non-Hispanic)	0.64	0.48–0.85	**<0.01**	0.53	0.38–0.74	**<0.01**
Geography (reference group: urban)	0.61	0.55–0.68	**<0.01**	0.56	0.50–0.62	**<0.01**
Sex (reference group: male)	0.98	0.88–1.09	0.73	1.04	0.92–1.16	0.54
Marital status (reference group: single/widowed)						
Married/significant other	2.11	1.90–2.36	**<0.01**	2.07	1.84–2.33	**<0.01**
Divorced, separated	0.90	0.78–1.03	0.13	0.89	0.77–1.03	0.13
Age at diagnosis (reference group: 35–44)						
18–24	0.36	0.27–0.49	**<0.01**	0.43	0.32–0.58	**<0.01**
25–34	0.92	0.78–1.08	0.32	0.93	0.79–1.10	0.39
45–54	0.79	0.70–0.90	**<0.01**	0.77	0.67–0.88	**<0.01**
55–65	0.64	0.56–0.73	**<0.01**	0.61	0.53–0.70	**<0.01**
Education (reference group: high school graduate)						
Undergraduate degree	2.33	1.17–4.62	**0.02**			
Graduate school or above	7.32	3.34–16.01	**<0.01**			

Note: Bold font indicates statistical significance (*p* < 0.05); OR = Odds ratio, CI = Confidence interval, *p* = significance level.

## Data Availability

Restrictions apply to the availability of these data. Data was obtained from the Arkansas Clinical Data Repository (AR-CDR) and are available from the authors with the permission of AR-CDR.
